# Effect of *PACAP/PAC1R* on Follicle Development of Djungarian Hamster (*Phodopus sungorus*) with the Variation of Ambient Temperatures

**DOI:** 10.3390/biology12020315

**Published:** 2023-02-15

**Authors:** Yan Qi, Huiliang Xue, Jinhui Xu, Ming Wu, Lei Chen, Laixiang Xu

**Affiliations:** School of Life Sciences, Qufu Normal University, Qufu 273165, China

**Keywords:** ambient temperature, *PACAP/PAC1R*, FSH, follicle development, *Phodopus sungorus*

## Abstract

**Simple Summary:**

Ambient temperature has affected the physiological activities of wild animals, such as reproduction. Pituitary adenylate cyclase-activating polypeptide (PACAP) and its receptor (PAC1R) regulate reproduction in mammals via influencing follicle development under ambient temperature variations. However, the effect of *PACAP/PAC1R* on the reproduction of *Phodopus sungorus* remains unclear. In this study, we explored the relationship between *PACAP/PAC1R* and follicle-stimulating hormone (FSH), involved in follicle development, at different ambient temperatures, which will ultimately influence the reproduction of *Phodopus sungorus*. The development of growing follicles and antral follicles were inhibited at low (8 °C, 14 °C) and high (29 °C) temperatures as well as *PACAP/PAC1R* expression and FSH serum concentration. The PKA/PKG and PKC phosphorylation sites of PACAP/PAC1R may be involved in the pathway of FSH synthesis through cAMP-PKA and its downstream signal pathway. Moreover, there was a significant positive correlation between the expression levels of *PACAP/PAC1R* and the number of the growing and antral follicles, as well as the serum FSH concentration and the number of antral follicles. In conclusion: (1) *PACAP/PAC1R* is evolutionarily conservative and exerts functions through major functional elements; (2) the temperature-dependent follicle development is correlated with the expression of *PACAP/PAC1R* and the serum FSH concentration. Therefore, *PACAP/PAC1R* and FSH are involved in the follicle development at different temperatures. These results not only provide a working basis for the study of *Phodopus sungorus* reproduction, but also provide a theoretical basis for the regulation of population dynamic equilibrium.

**Abstract:**

In *Phodopus sungorus*, the relationship between pituitary adenylate cyclase-activating polypeptide (PACAP) and its receptor (PAC1R), follicle-stimulating hormone (FSH), and follicle development remains unclear. In this study, we found that the development of growing follicles and antral follicles were inhibited at low (8 °C, 14 °C) and high (29 °C) temperatures. Meanwhile, *PACAP/PAC1R* expression and follicle-stimulating hormone (FSH) serum concentration significantly decreased during ambient temperatures of 8 °C, 14 °C and 29 °C compared to 21 °C. Thus, ambient temperature may influence the expression of *PACAP/PAC1R* and the synthesis of FSH for involvement in follicle development. Moreover, PACAP/PAC1R had major functional elements including PKA/PKG and PKC phosphorylation sites, which may involve in the pathway of FSH synthesis through cAMP-PKA and its downstream signal pathway. Moreover, there was a significant positive correlation between the expression levels of *PACAP/PAC1R* and the number of the growing and antral follicles, as well as the serum FSH concentration and the number of antral follicles. However, there was no significant correlation between the expression levels of *PACAP/PAC1R* and the serum FSH concentration, indicating a complicated pathway between *PACAP/PAC1R* and FSH. In conclusion, ambient temperature affects the expression of *PACAP/PAC1R* and the serum FSH concentration. The expression of *PACAP/PAC1R* and the serum FSH concentration are correlated with follicle development, which implies that they are involved in follicle development, which will ultimately influence the reproduction of *Phodopus sungorus*. This study can lay the foundation for future investigation on the regulation mechanism of reproduction in *Phodopus sungorus*.

## 1. Introduction

In order to adapt to the changing environment, seasonal breeding animals will adjust their reproductive activities to occur in a specific period. As an external environmental factor of seasonal reproduction, photoperiod regulates the structure and function of the female ovary via the regulation of the hypothalamus–pituitary–ovary (HPO) axis [1,2,3]. Follicle development plays a key role in female reproduction. When the duration of light is <12 h, it will interrupt the development of follicles and lead to ovarian degeneration [1,4]. However, when the illumination time is prolonged (>12 h), follicle development spans the preantral follicle stage, thus promoting follicle development [4,5]. In addition, multiple studies have shown that temperature is also involved in the seasonal reproduction of animals [6,7]. Global warming has led to a decline in the numbers of many wildlife species [8] and even to the extinction of some species, implying that ambient temperature could affect the population density of wildlife. It is not only high temperatures that are harmful, but lower temperatures can also inhibit or affect the reproductive cycle. Golden hamsters are known to be very sensitive to temperature fluctuations, as their gonadal activity decreases significantly at 5 ± 1 °C [9]. Ambient temperature is one of the critical factors affecting the animal’s seasonal reproduction [10]. Studies have shown that heat stress can inhibit ovarian follicle development and lead to ovarian dysfunction [11]. Therefore, ambient temperature could affect the seasonal reproduction of female animals by regulating follicle development.

Pituitary adenylate cyclase-activating polypeptide (PACAP) is a hypophysiotropic hormone originally discovered in the ovine hypothalamus [12], which is temperature-dependent [13,14]. PACAP binds to three receptor subtypes, PACAP type 1 receptor (PAC1R), vasoactive intestinal polypeptide type 1 receptor (VPAC1R) and VPAC2R, but PAC1R is the specific receptor of PACAP [15]. All receptors belong to the G protein-coupled receptor family and are expressed in various tissues. Later, PACAP and its specific receptor PAC1R were detected in rats [16] and mice [17], and the primary amino acid sequence of PACAP and PAC1R is highly conserved, suggesting that PACAP and PAC1R may play important roles in organisms [18]. A variety of important biological functions of PACAP have been found, including regulation of feed intake, stress, metabolism, and circadian rhythm [19,20,21]. PACAP mainly acts through PAC1R and stimulates the phospholipase C/protein kinase C (PKC)/calcium and adenylate cyclase/protein kinase A (PKA) pathways [12]. PAC1R mainly binds to Gαs protein, and induces the rapid production of cyclic adenosine monophosphate (cAMP), and finally activates PKA [22,23]. PKA catalyzes the hydroxyl phosphorylation of Ser/Thr residues of its target protein, resulting in biological effects [24]. In rat pituitary cells, PACAP can activate the mitogen-activated protein kinase (MAPK) pathway through the cAMP-PKA signaling pathway mediated by Gαs, and then induce FSH β [25,26]. In this pathway, PACAP uses the classical growth factor pathway to activate the expression of cFOS through MAPK phosphorylation of transcription factor ELK, and then activate FSH β [26]. Furthermore, PACAP and PAC1R may participate in the regulation of reproduction and follicle development via the HPO axis [27,28,29]. In studies, PACAP has been shown to inhibit FSH synthesis via the HPO axis to regulate reproduction [30]. It can also promote the development of antral follicles to the preovulatory stage by stimulating FSH and participating in the regulation of follicle development [31]. However, the effect of *PACAP/PAC1R* and FSH involved in follicle development of *Phodopus sungorus* under ambient temperature stress has not been studied.

Population dynamic equilibrium means that the population abundance is basically stable in the ecosystem [32]. The population abundance is determined by the intensity of animal reproductive activity. Djungarian hamster (*Phodopus sungorus*) is a seasonal breeder [33,34], which is one of the dominant rodents in the grasslands of Inner Mongolia [35]. *Phodopus sungorus* has a high reproductive capacity that begins in April and generally continues through September [36], and its average litter size is 5–6 [37]. In addition, its large relative body surface area, high metabolic rate, high body temperature, high thermal conductivity, narrow thermoneutral zone, and moderate ability to produce heat without shivering make it much more sensitive to fluctuations in ambient temperature [38,39]. Therefore, *Phodopus sungorus* was one of the most suitable samples for studying the follicle development regulated by the ambient temperature. Previous studies have shown that in male Siberian hamsters, different ambient temperatures have no significant effect on testicular weight and testicular FSH specific binding [40], but have significant effects on testosterone T3 and T4, thus affecting testicular function [41]. However, the effect of ambient temperature on the ovarian function of female *Phodopus sungorus* is not clear. In the present study, the functions of PACAP and PAC1R at different temperatures and their structural properties were investigated to confirm their roles in follicle development in *Phodopus sungorus*. The objectives of this study were to (1) assess the status of follicle development at different temperatures, (2) analyze the structural features of the complete sequence of the coding region of *PACAP/PAC1R*, (3) investigate the variations of *PACAP/PAC1R* expression levels at different ambient temperatures, (4) investigate the serum concentration of follicle-stimulating hormone (FSH) at different ambient temperatures, (5) analyze the correlation among the *PACAP/PAC1R* expression, FSH concentration in serum, and the number of growing follicles and antral follicles. These results provide a theoretical basis for uncovering the molecular mechanism of PACAP and PAC1R mediating the follicle development in wild animals at different temperatures. They not only provide a working basis for the study of *Phodopus sungorus* seasonal reproduction, but also provide a theoretical basis for the regulation of population dynamic equilibrium.

## 2. Materials and Methods

### 2.1. Sample Collection and Tissue Preparation

Adult females of *Phodopus sungorus* used in this study were captured in the field from Xilinhot, Inner Mongolia (N43°02′ E115°18′) in October. As this period, the filed ambient temperature is about 14 ± 3 °C and most of the *Phodopus sungorus* are in the non-breeding state. The captured rodents were identified, numbered, and fed in a feeding room at the experimental center of Qufu Normal University. The composite food particles used for feeding the rat were purchased from Jinan Peng Yue Experimental Animal Breeding co., Ltd. and applied with tap water ad libitum. The feeding room had adequate natural light, and the temperature was maintained at 21 ± 2 °C. All hamsters adapted in the feeding room for two weeks. All experiments were conducted in compliance with the rules of Qufu Normal University (Permit Number: 2020067) and the practicing rules of the China Ethics Committee for Experimental Animals.

Sixteen adult (2–3 months of age) female hamsters were selected by estimations of the degree of molar wear, which is a common indicator to identify the age of mammals, mainly rodents [42,43]. The weight of all the selected animals was 28 ± 1 g, and the deviation in weight among the selected animals was no more than 5%. Then, the selected individuals were randomly split into four different temperatures conditions including 8 °C, 14 °C (low temperatures), 21 °C (optimum temperature), and 29 °C (high temperature). Additionally, four female hamsters were included under each temperature condition. Then, four different temperatures were kept under moderate daylight (light:darkness = 12 h:12 h), 55% ± 5% relative humidity (RH), and submitted to the different temperature conditions for 4 weeks [44]. At 22:00 on the last day, all hamsters were sacrificed by carbon dioxide suffocation after staying in the dark for at least 2 h [45]. Immediately after that, fresh blood was collected, and serum was extracted. Ovaries were taken out and put into 4% paraformaldehyde. The hypothalamus was quickly removed, collected, and stored at −80 °C until further tests.

### 2.2. Microstructure Observation of Ovarian Follicles

After being fixed in 4% paraformaldehyde (G1101-500ML, Servicebio, Wuhan, China) fixative for 48 h, ovaries were washed, dehydrated, and then embedded in paraffin. Ovaries were cut into 5 micron slices. After H&E staining, the sections were sealed, and the ovarian tissue structure was photographed and observed under an optical microscope (Upright optical microscope, Eclipse E100, Nikon, Tokyo, Japan; Imaging system, DS-U3, Nikon, Tokyo, Japan). According to the classification criteria of growing follicles, antral follicles, and mature follicles, they are defined as follows: growing follicles have more than three layers of cubic granulosa cells and no antrum present [46]; antral follicles have multiple layers of granulosa cells and antrum present [1]; mature follicles are in the superficial layer of the ovarian cortex, and the follicular cavity is enlarged and filled with follicular fluid, which is round or oval in shape. The oocytes are located on one side of the follicle and form cumulus with the surrounding granulosa cells, and there are 3–4 layers of granulosa cells at the top of the cumulus [46]. The right ovaries of four individuals were used from each group and three sections of each ovary were examined. The average number of growing follicles and antral follicles per cross section were counted under the same magnification in each group.

### 2.3. Total RNA Extraction and RT-PCR

Total RNA was extracted from the hypothalamus of the sacrificed rodents using TRIzol reagent (D9108A, TaKaRa, Osaka, Japan). The concentration and the purity of total RNA were examined by the A260/A280 ratio using an ultraviolet spectrophotometer (Eppendorf, Hamburg, Germany). The integrity of total RNA was also checked using agarose gel electrophoresis. According to the instructions of TaKaRa RNA PCR Kit (AMV) 3.0, all RNA samples were reverse-transcribed using the AMV reverse transcriptase (2621, TaKaRa, Osaka, Japan) and an oligodeoxythymine (oligo(dT)_18_) (3806, TaKaRa, Osaka, Japan). All cDNAs obtained were stored at −80 °C.

### 2.4. Gene Cloning

Based on the *PACAP/PAC1R* cDNA sequences from *Cricetulus griseus* (*PACAP* cDNA sequence GenBank ID: NW_003614869.1 and *PAC1R* cDNA sequence GenBank ID: NW_003614013.1), which is evolutionarily similar to those of *Phodopus sungorus*, the primers were designed using Primer 5 and Oligo 7 (Tables S1 and S2). The PCR reaction system was 25 µL in volume and was composed of 14.3 µL ddH_2_O, 2.5 µL 10 × PCR buffer (Mg^2+^ Free), 2.0 µL MgCl_2_ (25 mmol/L), 2.0 µL dNTP mixture (2.5 mmol/L), 1.0 µL forward primer, 1.0 µL reverse primer, 0.2 µL TaKaRa Taq (5 U/µL), and 2.0 µL cDNA template. The PCR reaction procedures were as follows: (1) pre-denaturation at 94 °C for 5 min, (2) denaturation at 94 °C for 1 min, (3) annealing for 1 min (annealing temperatures are listed in Tables S1 and S2), (4) extension at 72 °C for 1 min. Steps 2–4 were repeated for 35 cycles, and (5) final extension took place at 72 °C for 10 min. The products of PCR amplification were detected using a 1.5% agarose gel electrophoresis (AGE) and purified using the DNA Gel Extraction Kit (TSP601-50, Tsingke Biotechnology Co., Ltd., Qingdao, China). The purified product was then connected to a PMD19-T vector (6013, TaKaRa, Osaka, Japan) and transformed into Escherichia coli DH5α competent cells. Finally, 8 positive clones were obtained and sequenced at San bo Yuan Zhi Co., Ltd. in Beijing, China.

### 2.5. Real-Time Fluorescence Quantitative PCR

All real-time fluorescence quantitative PCR reactions were performed using the Qiagen Rotor-Gene Q Platform (QIAGEN, Hilden, North Rhine-Westphalia, Germany) with SYBR^®^ Green Premix HS qPCR Kit II (AG11702, Accurate Biotechnology (Hunan) Co., Ltd., Changsha, China). The specific primers for real-time quantitative PCR were designed based on amplified target sequences and mouse *β-actin*. These primers were synthesized by Sanbo Yuanzhi Co., Ltd., Beijing, China. The reaction volume was as follows: 7.2 µL DEPC H_2_O, 10 µL SYBR Green, 0.4 µL forward primer and reverse primer (10 µmol/L), and 2 µL cDNA template. The initial polymerase chain reaction was activated at 94 °C for 5 min, followed by 40 cycles, which included the following steps: 94 °C for 1 min, annealing for 45 s (annealing temperatures are listed in Table S3), and 72 °C for 70 s. Fluorescent signals were collected after the elongation step of each PCR cycle. The integrity of the product was tested by 1.5% AGE, and a fusion curve with a single peak confirmed a unique amplification. The amplification efficiency of these gene-specific primers was between 90% and 110%, and the fitting degree exceeded 0.99, as confirmed by the standard curve test [47]. The quantity of *PACAP* mRNA and *PAC1R* mRNA were shown in the 2^−△△CT^ way (normalization to *β-actin* first, and then compared with control group) [48].

### 2.6. FSH Hormone Content Determination

After the fresh blood was collected and the serum was extracted from *Phodopus sungorus*, it was immediately transferred into a 1.5 mL Eppendorf centrifuge tube, which was placed at 4 °C in a refrigerator for 30 min and then centrifuged at 3000 rpm for 15 min. Following this, the serum in the upper layer of the 1.5 mL Eppendorf centrifuge tube, in a light-yellow transparent shape, was transferred into a new 1.5 mL Eppendorf centrifuge tube. The serum concentrations of FSH were determined by enzyme-linked immunoassay according to the kit instructions (ELISA, Shanghai Hengyuan Biological Co. HB020-Hr, Shanghai, China). In the experiment, a blank well control (only sample dilution), standard well (standard dilution diluted by different gradient multiple), and tested sample wells were set up. Then, 50 μL detection solutions (40 μL diluents and 10 μL supernatants) were added to every tested sample well. After being incubated for 20 min at 37 °C and washed, the standard well and the tested sample well were added with 50 μL of enzyme-labeled reagent. After being re-incubated and washed, the blank well, standard well, and tested sample well were added with 50 μL chromogenic agent A and 50 μL chromogenic agent B and placed in the dark environment with 37 °C for 10 min. Finally, 50 μL terminating solution was added to each well. Each sample absorbance was assessed at 450 nm with microplate reader (SynergyH1, Bio-RAD, Hercules, CA, USA). Then, the concentration of FSH in serum was calculated according to the standard curve.

### 2.7. Statistical Analysis

Signal peptides of PACAP/PAC1R were predicted using SignalP 4.0 (http://www.cbs.dtu.dk/services/SignalP/ (accesses on 15 December 2021) [49], and transmembrane region was predicted using the TMHMM 2.0 (http://www.cbs.dtu.dk/services/TMHMM-2.0/ (accesses on 8 January 2022) [50]. Amino acid functional sites were predicted using the PredictProtein (http://www.predictprotein.org) [51] and Prosite (http://www.expasy.ch/prosite/ (accesses on 20 January 2022) [52]. Shapiro–Wilk and Levene were performed to test normality of the data and homogeneity of variances. Using SPSS Statistics 22.0, the number of growing follicles and antral follicles, the expression levels of *PACAP/PAC1R* mRNA in the hypothalamus, and the serum concentrations of FSH at 8 °C, 14 °C, 21 °C, and 29 °C were analyzed by the one-way analysis of variance (ANOVA) test, and Fisher’s least significant difference (LSD) back testing. Correlation among the expression levels of *PACAP/PAC1R*, the serum concentrations of FSH, and the number of growing follicles and antral follicles were analyzed by GraphPad Prism v8. Data were expressed as means ± standard error of the mean (SEM). *p*-value < 0.05 was considered significant.

## 3. Results

### 3.1. Differences in the Number of Growing Follicles and Antral Follicles at Different Temperatures

**Figure 1:**
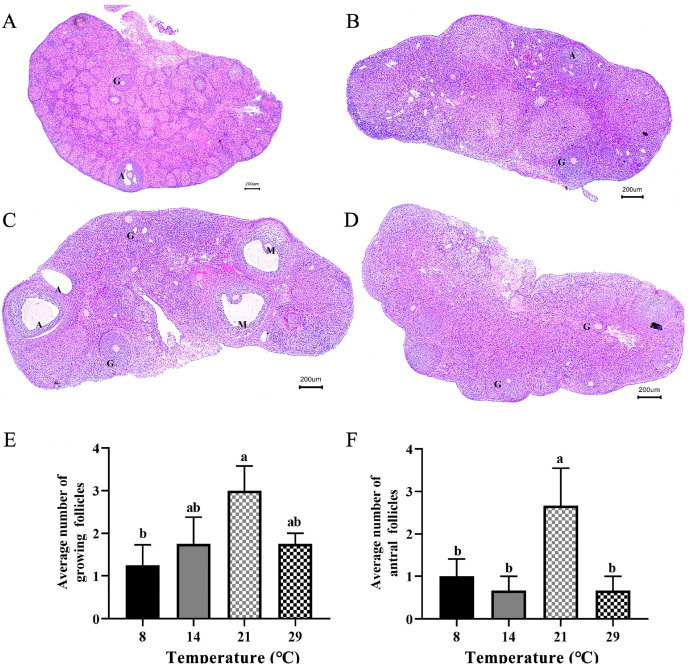
Differences in the number of follicles by HE staining of ovarian section in *Phodopus sungorus* at 8 °C, 14 °C, 21 °C, and 29 °C. (**A**) Ovarian section of individuals from 8 °C. (**B**) Ovarian section of individuals from 14 °C. (**C**) Ovarian section of individuals from 21 °C. (**D**) Ovarian section of individuals from 29 °C. (**E**) Differences in the average number of growing follicles on per cross section under different temperatures. (**F**) Differences in the average number of antral follicles on per cross section under different temperatures. G, growing follicle; A, antral follicle; M, mature follicle. Values are means ± SEM. Bar = 200 μm; n = 4. Different letters above the columns indicate significant differences (*p* < 0.05).

Figure 1A–D depict the structures of follicles in the ovaries of *Phodopus sungorus* at different temperatures. In Figure 1C, it is worth noting that mature follicles can be found in the optimal temperature (21 °C), but not in the other temperatures. In Figure 1E, the average number of growing follicles per cross section was significantly higher (*p* < 0.05) at the optimum temperature (21 °C) than at low temperature (8 °C). Meanwhile, the average number of antral follicles per cross section was significantly higher (*p* < 0.05) at the optimum temperature (21 °C) than at low (8 °C, 14 °C) and high (29 °C) temperatures (Figure 1F).

### 3.2. Characterization of PACAP and PAC1R

Two specific nucleotide fragments (421 bp and 216 bp) for *PACAP* and three specific fragments (619 bp, 915 bp, and 429 bp) for *PAC1R* were obtained by PCR (Figures S1 and S2). A 574 bp cDNA fragment was assembled for *PACAP*, which included 40 bp of 5’-UTR and 534 bp of the complete coding sequence, encoding 177 amino acid residues (GenBank ID: OK337681). A 1463 bp cDNA sequence of *PAC1R* was also assembled, which included a 1404 bp complete coding sequence encoding 467 amino acids and 59 bp of 3’-UTR (GenBank ID: OK337682).

**Figure 2:**
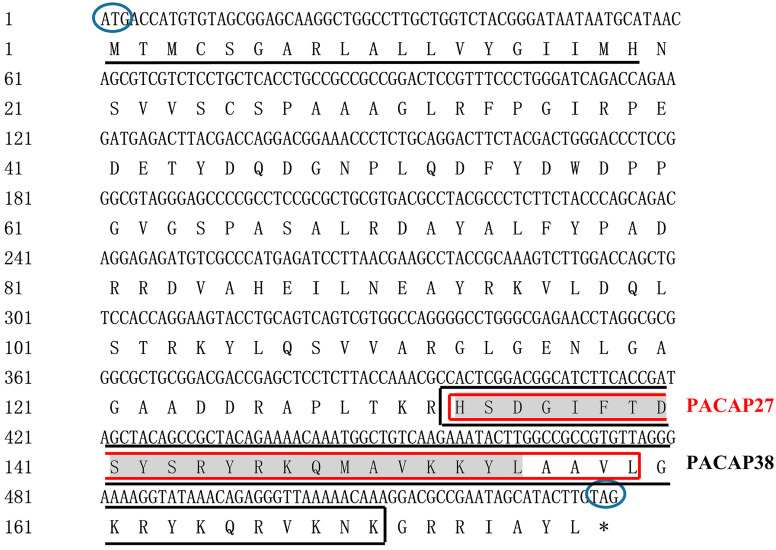
Nucleotide sequence and the derived amino acid sequence of the coding region of *PACAP*: The number of nucleotides and derived amino acid residues are shown on the left. The two polypeptides PACAP27 and PACAP38 are represented by the box. Oval indicates the start and stop codons. Underlined are the signal peptides and shading represents the glucagon/GIP/secretagogue/VIP family marker. * indicates that the stop codon (TGA) does not encode amino acids.

**Figure 3:**
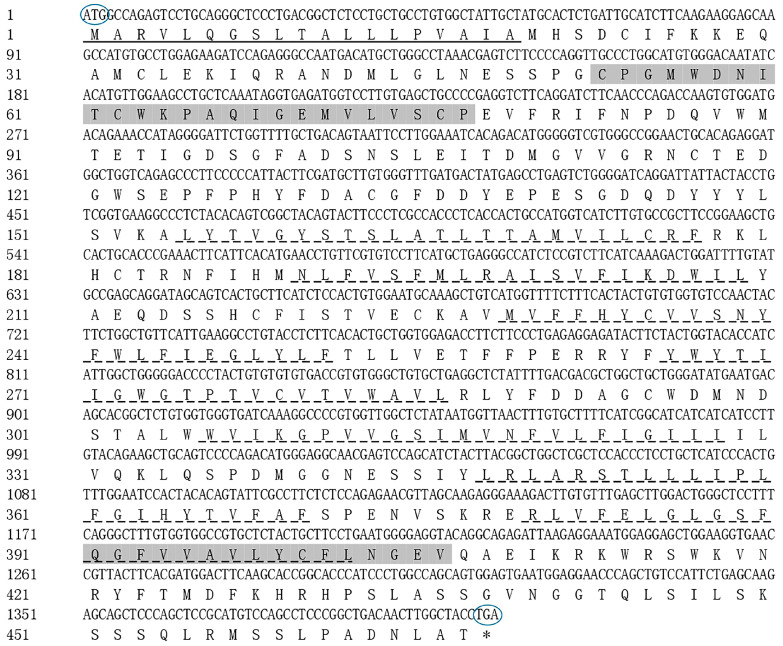
Nucleotide sequence and derived amino acid sequence of coding region of *PAC1R*: The number of nucleotides and derived amino acid residues are shown on the left. Oval indicates start and stop codons. Underlined are the signal peptides. The dotted line marks 7 transmembrane regions and the G protein-coupled receptor family tag in the shaded portion. * indicates that the stop codon (TGA)does not encode amino acids.

Two predicted PACAP polypeptides (PACAP38 and PACAP27) were obtained using the *PACAP* nucleotide sequence (GenBank ID: OK337681) of *Phodopus sungorus*, and which were consistent with the earlier results [23]. The amino acid sequences of PACAP38 and PACAP27 were HSDGIFTDSYSRYRKQMAVKKYLAAVLGKRYKQRVKNK-NH2 and HSDGIFTDSYSRYRKQMAVKKYLAAVL-NH2, respectively. Both PACAP38 and PACAP27 had the tag sequence of the Glucagon/GIP/Secretin/VIP family (Figure 2). A signal peptide was also detected in the PACAP polypeptide (Figure 2), which is characteristic of a secreted transmembrane protein, confirming that PACAP belongs to neurotransmitters, neuromodulators, and neurotrophic factors [53]. In addition, PACAP has several post-translational modification sites, including PKA/PKG phosphorylation site and PKC phosphorylation sites, N-cardamom acylation site and amidation sites (Table 1). Phosphorylation sites are essential for proteins and their transport and function, in which PKA/PKG and PKC phosphorylation sites can phosphorylate serine and threonine residues, thus giving full play to the biological function of proteins [54]. We found that PACAP27 and PACAP38 both have the PKA/PKG phosphorylation site and amidation sites. Meanwhile, the amino acid sequence of PAC1R was predicted using the PAC1R nucleotide sequence (GenBank ID: OK337682) obtained from *Phodopus sungorus*; seven transmembrane regions, and the GPCRs family tag were detected in the predicted amino acid sequence of PAC1R (Figure 3), which confirmed that PAC1R belongs to the GPCRs family [55]. In addition, PAC1R also has several post-translational modification sites, including PKC phosphorylation sites, N-glycosylation site, Tyrosine kinase II phosphorylation site, and N-glycosylation site (Table 2).

### 3.3. Differential mRNA Expression Levels of PACAP and PAC1R in the Hypothalamus of Female Phodopus Sungorus at Different Ambient Temperatures

**Figure 4:**
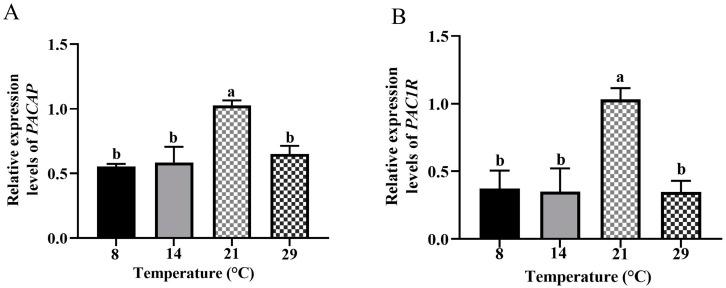
Comparations of the expression levels of *PACAP* (**A**) and *PAC1R* (**B**) in the hypothalamus of *Phodopus sungorus* at 8 °C, 14 °C, 21 °C, and 29 °C. Data are expressed as means ± SEM. n = 4. Different letters above the columns indicate significant differences (*p* < 0.05 or *p* < 0.01).

The difference in expression levels of *PACAP* and *PAC1R* was quantitatively demonstrated for female *Phodopus sungorus* at different temperatures (Figure 4). Through 2^−△△CT^, the expression level of *PACAP* at low temperatures (8 °C, *p* < 0.01; 14 °C, *p* < 0.05) was significantly lower than that at the optimum temperature (21 °C), and was also lower at high temperature (29 °C, *p* < 0.01). Furthermore, the expression level of *PAC1R* at low (8 °C, 14 °C) and high (29 °C) temperatures was also significantly lower (*p* < 0.01) than at the optimum temperature (21 °C). Therefore, the expression levels of *PACAP* and *PAC1R* were significantly decreased in both low and high-temperature groups indicating that the expression of *PACAP* and *PAC1R* in the hypothalamus of female *Phodopus sungorus* is greatly affected by ambient temperature.

### 3.4. The Serum Concentration of FSH

**Figure 5:**
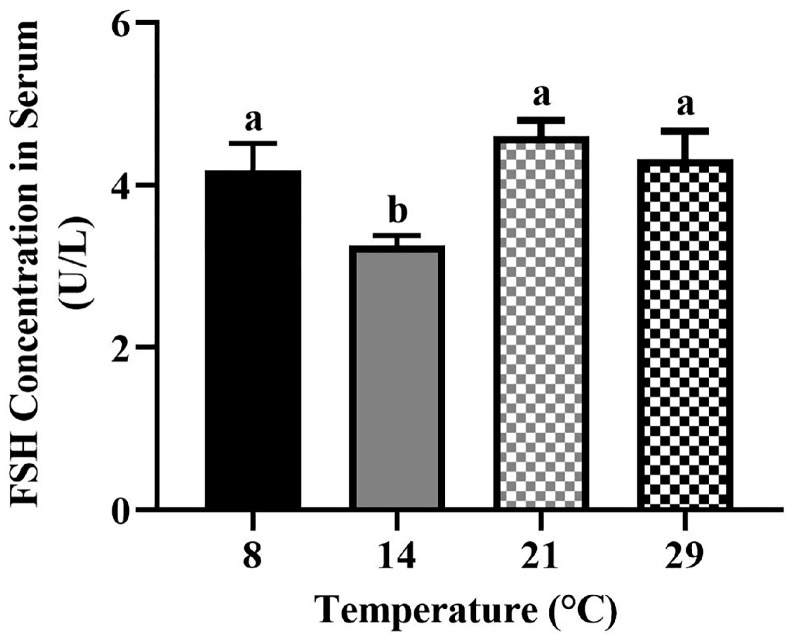
Comparations of the FSH concentration in serum of *Phodopus sungorus* at 8 °C, 14 °C, 21 °C, and 29 °C. Data are expressed as means ± SEM. n = 4. Different letters above the columns indicate significant differences (*p* < 0.05).

The serum concentration of FSH was the highest in the optimum temperature (21 °C) and decreased in the low (8 °C, 14 °C) and high (29 °C) temperatures (Figure 5). The concentrations of FSH at 8 °C, 21 °C, and 29 °C were significantly higher (*p* < 0.05) than the FSH concentration at 14 °C. However, there was no significant difference (*p* > 0.05) between 8 °C, 21 °C and 29 °C.

### 3.5. Analysis of Correlation between the PACAP/PAC1R Expression and the Number of Growing Follicles and Antral Follicles

**Figure 6:**
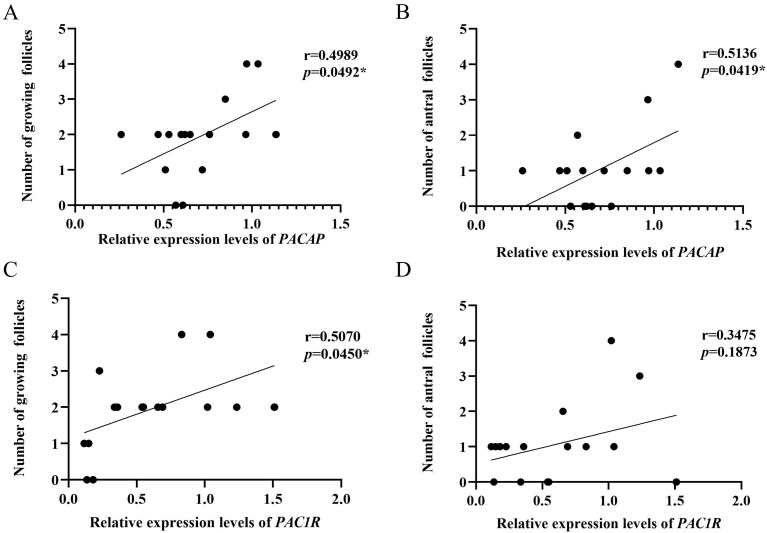
Correlation analysis between the *PACAP/PAC1R* expression and growing follicles number and antral follicles number in *Phodopus sungorus*. (**A**) Correlation analysis between *PACAP* expression and growing follicles number. (**B**) Correlation analysis between *PACAP* expression and antral follicles number. (**C**) Correlation analysis between *PAC1R* expression and growing follicles number. (**D**) Correlation analysis between *PAC1R* expression and antral follicles number (* *p* < 0.05).

The expression levels of *PACAP* were positively correlated with the number of growing follicles (r = 0.4989, *p* = 0.0492) and antral follicles (r = 0.5136, *p* = 0.0419; Figure 6A,B); while, the expression level of *PAC1R* was only positively correlated (r = 0.5070, *p* = 0.0450) with the number of growing follicles, but not with antral follicles (r = 0.3475, *p* = 0.1873; Figure 6C,D). Therefore, the expression levels of *PACAP/PAC1R* are correlated with follicle development.

### 3.6. Analysis of Correlation between FSH Concentration in Serum and the Number of Growing Follicles and Antral Follicles

**Figure 7:**
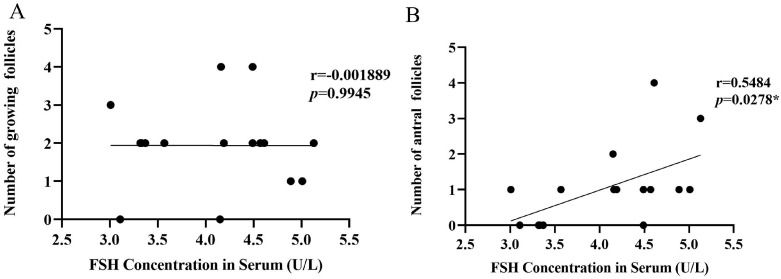
Correlation analysis between the serum FSH concentration and growing follicles number and antral follicles number in *Phodopus sungorus*. (**A**) Correlation analysis between serum FSH concentration and growing follicles number. (**B**) Correlation analysis between serum FSH concentration and antral follicles number (* *p* < 0.05).

The serum concentration of FSH was not correlated with the number of growing follicles (r = −0.001889, *p* = 0.9945; Figure 7A), but was positively correlated with the number of antral follicles (r = 0.5484, *p* = 0.0278; Figure 7B). Therefore, the serum concentration of FSH is correlated with follicle development.

### 3.7. Analysis of Correlation between the PACAP/PAC1R Expression and FSH Concentration in Serum

**Figure 8:**
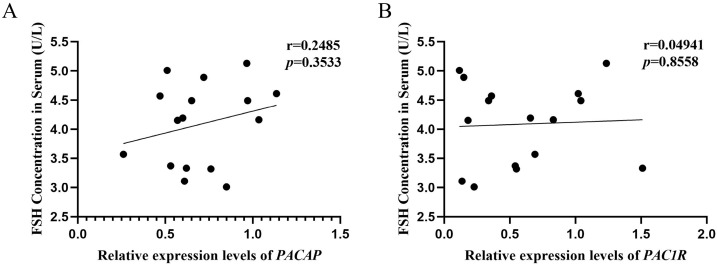
Correlation analysis between the *PACAP/PAC1R* expression and serum FSH concentration in *Phodopus sungorus*. (**A**) Correlation analysis between *PACAP* expression and serum FSH concentration. (**B**) Correlation analysis between *PAC1R* expression and serum FSH concentration. *p* < 0.05 represents significant difference.

The expression levels of *PACAP* (r = 0.2485, *p* = 0.3533) and *PAC1R* (r = 0.04941, *p* = 0.8558) were not correlated with the serum concentration of FSH (Figure 8A,B).

## 4. Discussion

Ambient temperature and photoperiod have seriously affected the population abundance of many species [56]. The ovary is the primary organ of female reproductive activity, and its structure and function are critical for reproduction regulation. The stage of follicle development is critical for the function of the ovary [57]. At different ambient temperatures, the photoperiod <12 h inhibited reproductive activity while >12 h activated reproductive activity [40], indicating that 12 h may be the time for the *Phodopus sungorus* to start reproduction. Moreover, some scholars have carried out related research by setting the same photoperiod (light:darkness: = 12 h:12 h) [58], which can provide the method guidance of our study. Therefore, we set different ambient temperatures in the same photoperiod (light:darkness: = 12 h:12 h) in order to better explore the effect of temperature on ovarian follicle development. In this study, we discovered that the number of growing follicles and antral follicles was highest in the optimum temperature, and mature follicles appeared. Meanwhile, in both the low and high temperatures, low numbers of antral follicles were present as opposed to no mature follicles (Figure 1), indicating that the optimum temperature can promote follicle development, while both high and low temperatures can inhibit follicle development. This finding is consistent with what has been observed in sheep [59]. The hypothalamus and pituitary regulate follicle development [60], and PACAP/PAC1R is a key reproductive regulator in the hypothalamus [61]. We found that after 4 weeks of different temperature treatments, the expression levels of *PACAP/PAC1R* were significantly reduced at low and high temperatures, compared to the optimum temperature (Figure 4). This result is somewhat similar to that found in female blue gourami (*Trichogaster trichopterus*), where both low and high-temperature groups had significantly reduced expression of *PACAP/PAC1R* [62]. Furthermore, the concentration of serum FSH was temperature dependent, meaning that, when compared to the optimum temperature group, the concentration of low and high temperature hormones decreased (Figure 5). It is worth noting that the expression of *PACAP/PAC1R* and the serum concentration of FSH hormone correspond to the follicle development trend. This is consistent with the fact that PACAP can initiate immature follicles and cause their antral follicles to escape apoptosis when stimulated by FSH, allowing them to enter the preovulatory stage [31].

Several researches have revealed that the conservative phosphorylation sites existing in PACAP/PAC1R can promote biological function and regulate biological processes [63,64,65]. In our study, compared with rats [66], rabbits [67], and humans [68], the result of sequence analyses of *PACAP*/*PAC1R* indicated that it was highly conservative (Figure 2), and there were two active forms of these peptides, HSDGIFTDSYSRYRKQMAVKKYLAAVLGKRYKQRVKNK-NH2 (PACAP27) and HSDGIFTDSYSRYRKQMAVKKYLAAVL-NH2 (PACAP38). The evolutionary conservation of *PACAP* and *PAC1R* implies the significance of their biological function. Protein dephosphorylation and phosphorylation are critical for intracellular signal transduction and can regulate a variety of cellular processes [69]. In order to investigate the potential mechanism of *PACAP/PAC1R* and FSH involved in follicle development in *Phodopus sungorus*, we looked at the *PACAP/PAC1R* coding region sequence and post-translational modification site. PACAP has several phosphorylation sites, including the PKA/PKG phosphorylation site and PKC phosphorylation site, which we discovered (Table 1). PKA/PKG phosphorylation sites are found in both PACAP27 and PACAP38. Studies have shown that PKA can phosphorylate the serine/threonine residues in substrate protein or enzyme molecules, thereby exerting biological functions, and its activity is regulated by cAMP, and it participates in follicle regulation as well [70,71,72]. PACAP can activate the intracellular signaling pathways PKA and PKC [73]. This may be the main regulatory pathway of PACAP involved in the follicle development. Furthermore, PAC1R also possessed a GPCRs family tag, indicating that it belonged to GPCRs with seven transmembrane domains (Figure 3). PAC1R also has several phosphorylation sites, including PKC and Tyrosine kinase II phosphorylation sites. Furthermore, PAC1R has a N-cardamom acylation site and N-glycosylation sites (Table 2), which are closely related to material transport and PAC1R protein localization [74,75]. PACAP has been discovered to act via GPCRs. PACAP primarily stimulates the adenylate cyclase/cAMP pathway. The activation of its receptors (PAC1R) via this pathway leads to the activation of PKA and downstream pathways [76]. Meanwhile, FSH, as a coordinating factor of follicle development, can be directly regulated by PACAP, which promotes its release [77], and initiates signal transduction in the later stage of follicle development [78,79,80]. The action of high levels of FSH promotes follicle recruitment, the growth of primary follicles, and the maturation of follicles [81]. The primary signal transduction generated by FSH binding to cumulus-granulosa cells is thought to be regulated by cAMP-PKA [82]. However, some research suggests that PKC may also play a key role in FSH signal transduction [83]. Furthermore, studies have shown that the PACAP-induced cAMP pathway activates protein kinase (MAPK), stimulates cFOS, a necessary and sufficient key transcription factor for FSH β induction, induces FSH β subunit expression, and increases FSH concentration [26]. Therefore, the post-translational modification site of *PACAP/PAC1R*, especially PKA and PKC phosphorylation sites, may be involved in follicle development via changing FSH concentration at ambient temperature.

In order to explore the mechanism of how *PACAP/PAC1R* is involved in follicle development, correlation analyses were performed. *PACAP* expression was positively correlated with the number of growing follicles and antral follicles (Figure 6A,B), and *PAC1R* was also positively correlated with the number of growing follicles (Figure 6C,D). Those results indicated that *PACAP/PAC1R* was commonly correlated with the follicle development, which is consistent with previous findings that PACAP/PAC1R is involved in regulating follicle development [27]. However, in this study, we found that *PACAP/PAC1R* is state-dependent for the follicle development. Moreover, we found that FSH was positively correlated with the number of antral follicles, but not with growing follicles (Figure 7). This is because growing follicles are gonadotropin (FSH) insensitive, whereas antral follicle development is dependent on gonadotropin [84]. Therefore, hormones select dominant follicles to develop into preovulatory follicles, promoting the development of antral follicles [81,85]. In summary, at different temperatures, *PACAP/PAC1R* is correlated with different levels of follicle development, which may occur via change in the serum concentration of FSH. However, in this pathway, we found that there was no correlation between the expression of *PACAP/PAC1R* and the concentration of FSH in serum (Figure 8). Studies have shown that PACAP/PAC1R can also inhibit the secretion of FSH through the HPO axis [30]. For example, PACAP can stimulate the transcription of follistatin (Fst) and then inhibit the secretion of FSH [86]. This result indicates that the relationship may be complicated between PACAP/PAC1R and the synthesis of FSH, and needs to be studied further. These findings indicate that understanding the ambient temperature is critical for regulating the reproductive mechanism in animals and the dynamic balance of the population.

## 5. Conclusions

When the ambient temperature changes, the *PACAP/PAC1R* of mammals is correlated with changes in follicle development, and thus influences mammals’ reproduction. The potential influence of PACAP/PAC1R on follicle development in *Phodopus sungorus* under ambient temperature regulation was investigated in this study. The development of follicles at different temperatures was significantly different. *PACAP/PAC1R* is evolutionarily conservative and functions through major functional elements. Furthermore, the temperature-dependent follicle development is correlated with the expression of *PACAP/PAC1R* and the serum FSH concentration. Therefore, *PACAP/PAC1R* and FSH are involved in the follicle development at different temperatures. The results of this study can not only enrich the reproductive mechanism of rodents, but they also lay a rich theoretical foundation for exploring how to maintain the population dynamic balance of *Phodopus sungorus*.

## Figures and Tables

**Table 1:** Post-translational modification site of PACAP.

Post-Translational Modification Site	Modified Position	Amino Acid Sequence
PKA/PKG phosphorylation sites	131–134	KRHS
PKC phosphorylation sites	101–103	STR
	130–132	TKR
N-cardamom acylation site	6–11	GARLAL
	63–68	GSPASA
	113–118	GLGENL
	119–124	GAGAAD
amidation sites	159–162	LGKR
	170–173	KGRR

PKA, Protein Kinase A; PKG, Protein Kinase G; PKC, Protein Kinase C.

**Table 2:** Post-translational modification site of PAC1R.

Post-Translational Modification Site	Modified Position	Amino Acid Sequence
N-glycosylation site	47–50	NESS
	59–62	NITC
	116–119	NCTE
	299–302	NDST
	342–345	NESS
	374–377	NVSK
PKC phosphorylation sites	151–153	SVK
	376–378	SKR
	416–418	SWK
Tyrosine kinase II phosphorylation site	75–78	SCPE
	93–96	TIGD
	221–224	STVE
	376–379	SKRE
N-cardamom acylation site	45–50	GLNESS
	55–60	GMWDNI
	340–345	GGNESS
	362–367	GIHYTV
	388–393	GSFQGF
	438–443	GVNGGT

PKC, Protein Kinase C.

## Data Availability

The *PACAP/PAC1R* sequence data have been submitted to the GenBank databases. The original contributions presented in the study are included in the supplementary material; further inquiries can be directed to the corresponding author/s.

## Supplementary Materials

The following supporting information can be downloaded at https://www.mdpi.com/article/10.3390/biology12020315/s1, Table S1: The primers for *PACAP*. Table S2: The primers for *PAC1R*. Table S3: Fluorescence quantitative primers for *PACAP/PAC1R*. Figure S1: RT-PCR amplification of *PACAP* from *Phodopus sungorus.* Figure S2: RT-PCR amplification of *PAC1R* from *Phodopus sungorus.*
