# Supplementary material for: Effect of PACAP/PAC1R on Follicle Development of Djungarian Hamster (Phodopus sungorus) with the Variation of Ambient Temperatures
Source: Biology (Basel). 2023 Feb 15;12(2):315. doi: 10.3390/biology12020315 (PMC9953326; doi:10.3390/biology12020315)
Supplement: Supplementary file 1 [file biology-12-00315-s001.zip › biology-2154547-supplementary.pdf]

Supplementary Materials

Table S1. The primers for *PACAP*

Primer	Primer sequences	Product Length (bp)	Annealing temperature (°C)
Primer 1	F:5'–ATCAGACCAGAAGACGAGGCT–3′<br>R:5'–AGTATGCTATTCGGCGTCCTT–3′	421	58.5
Primer 2	F:5'–CGCTGTCCTACTTAGTCAACC–3′<br>R:5'–GGGTCCCAGTCATAGAAGTC–3′	216	60.0

Table S2 The primers for *PAC1R*

Primer	Primer sequences	Product Length (bp)	Annealing temperature (°C)
Primer 1	F:5'–CTCCTGCTGCCTGTGGCTATT–3′<br>R:5'–AGTGACTGCTGTCCTGCTCGG–3′	619	63.5
Primer 2	F:5'–AGGTGAGATGGTCCTTGTGAG–3′<br>R:5'–TCTGGAGAGAAGGCGAATACT–3′	915	58.5
Primer 3	F:5'–CATCTACTTACGGCTGGCTCG–3′<br>R:5'–AGAGGGGAGAGGCACAAACAT–3′	429	60.5

Table S3 Fluorescence quantitative primers for *PACAP/PAC1R*

Primer	Primer sequences	Product Length (bp)	Annealing temperature (°C)
PACAP	F:5'-CCTTGCTGGTCTACGGGATAA-3'<br>R:5'-GTTTCCGTCCTGGTCGTAAGT-3'	119	59.6
PAC1R	F:5'-CCAAGTGTGGATGACAGAAACC-3'<br>R:5'-CCTTCACCGACAGGTAGTAATAATC-3'	203	60.1

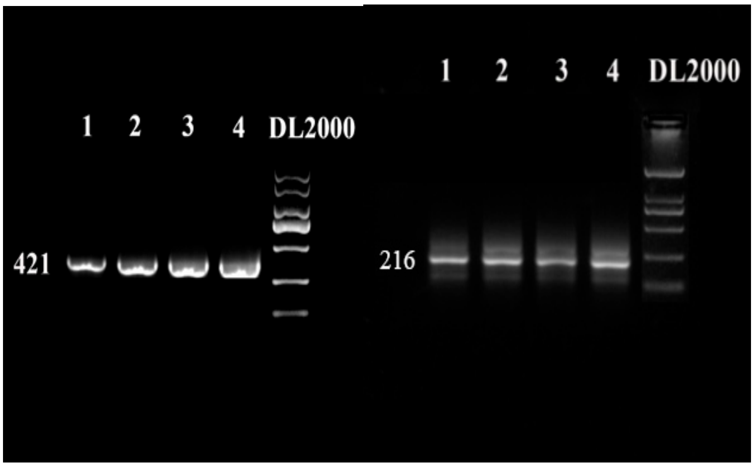

Figure S1. RT-PCR amplification of *PACAP* from *Phodopus sungorus*.

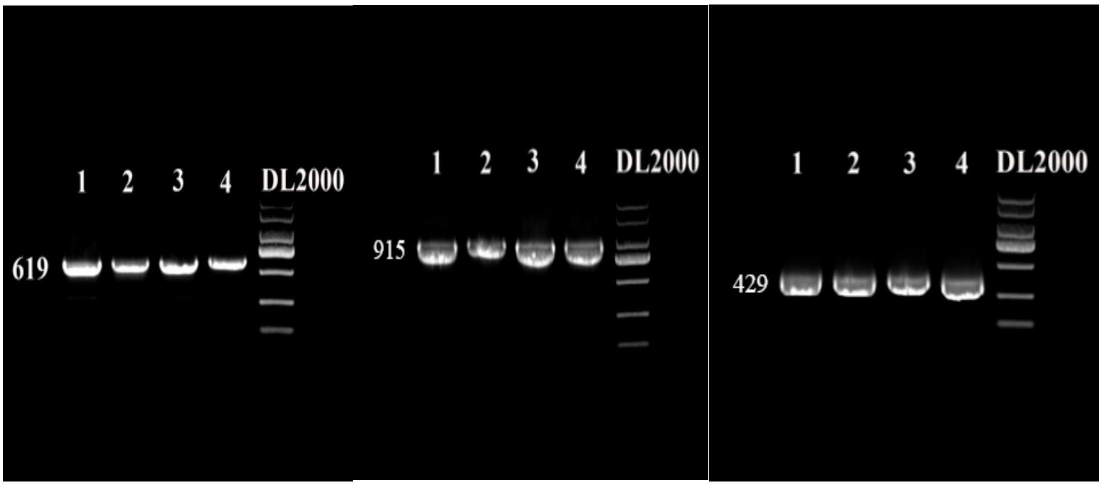

Figure S2. RT-PCR amplification of *PAC1R* from *Phodopus sungorus*.
